# Supplementary material for: Patient-Reported Postoperative Pain and Numbness: Applications for Endoscopic vs. Microscopic Ear Surgery
Source: J Pers Med. 2022 Oct 14;12(10):1718. doi: 10.3390/jpm12101718 (PMC9604827; doi:10.3390/jpm12101718)
Supplement: Supplementary file 1 [file jpm-12-01718-s001.zip › Supplemental Material File S1.pdf]

COLUMBIA | OTOLARYNGOLOGY HEAD AND NECK SURGERY

Ear Surgery Questionnaire

What is your age? _____

What is your gender? *Man* *Woman* *Other*

What problem are you here for (you may leave blank)? ________________________________

Please answer the following questions as accurately as possible:

Pretend you are a patient who is about to have ear surgery. On a scale from 0 (not important) to 10 (very important), how important are the following to you when you have ear surgery?

	← *Not important*									*Very important* →
Visibility of the scar	0	1	2	3	4	5	6	7	8	9	10
Cost of surgery to the hospital (not to you)	0	1	2	3	4	5	6	7	8	9	10
Pain control	0	1	2	3	4	5	6	7	8	9	10
Time spent under anesthesia	0	1	2	3	4	5	6	7	8	9	10
Hearing	0	1	2	3	4	5	6	7	8	9	10
An incision hidden in the ear canal (no outside scar)	0	1	2	3	4	5	6	7	8	9	10
Not having numbness of your ear	0	1	2	3	4	5	6	7	8	9	10
Color of the bandage you get after surgery	0	1	2	3	4	5	6	7	8	9	10
Size of the incision	0	1	2	3	4	5	6	7	8	9	10
Friendliness of staff	0	1	2	3	4	5	6	7	8	9	10
