# Supplementary material for: Patient-Reported Postoperative Pain and Numbness: Applications for Endoscopic vs. Microscopic Ear Surgery
Source: J Pers Med. 2022 Oct 14;12(10):1718. doi: 10.3390/jpm12101718 (PMC9604827; doi:10.3390/jpm12101718)
Supplement: Supplementary file 1 [file jpm-12-01718-s001.zip › Supplemental Material File S2.pdf]

Cuestionario de Cirugía de Orejas

¿Cual es tu edad? ______
¿Cuál es su género? Hombre Mujer Otro
¿Para qué problema estás aquí (puedes dejarlo en blanco)?
____

Por favor responda las siguientes preguntas con la mayor precisión posible:

Imagina que eres un paciente que está a punto de someterse a una cirugía de oído. En una escala de 0 (no importante) a 10 (muy importante), ¿qué tan importante son para usted las siguientes cosas al someterse a una cirugía de oído?

	← No importante										*Muy importante →*
Visibilidad de la cicatriz	0	1	2	3	4	5	6	7	8	9	10
Costo de la cirugía en el hospital (no para usted)	0	1	2	3	4	5	6	7	8	9	10
Control de dolor	0	1	2	3	4	5	6	7	8	9	10
Tiempo pasado bajo anesthesia	0	1	2	3	4	5	6	7	8	9	10
Audición	0	1	2	3	4	5	6	7	8	9	10
Una incisión oculta en el canal auditivo (sin cicatriz externa)	0	1	2	3	4	5	6	7	8	9	10
No tener adormecimiento de la oreja	0	1	2	3	4	5	6	7	8	9	10
Color del vendaje que se obtiene después de la cirugía	0	1	2	3	4	5	6	7	8	9	10
Tamaño de la incisión	0	1	2	3	4	5	6	7	8	9	10
Amabilidad del personal	0	1	2	3	4	5	6	7	8	9	10
